# AGE INCLUSIVITY DOMAINS OF HIGHER EDUCATION (AIDHE): A MODEL TO SUPPORT AGE-DIVERSE STUDENTS, FACULTY, AND STAFF

**DOI:** 10.1093/geroni/igae098.1431

**Published:** 2024-12-31

**Authors:** Joann Montepare, Nina Silverstein, Cal Halvorsen

**Affiliations:** Chair; Co-Chair; Discussant

## Abstract

The Age Inclusivity Domains of Higher Education (AIDHE) model (adopted in 2023 by GSA and its Academy for Gerontology in Higher Education) offers a guiding framework for how institutions of higher education can assess age-inclusive practices that impact students, faculty, and staff across seven domains of institutional function. In this symposium, presenters describe campus practices in three high-need, high impact domains: Teaching and Learning, Personnel, and Student Affairs. To begin, Bowen and colleagues (UMass Boston/Lasell University) will describe the AIDHE model and research using the Age-Friendly Inventory and Campus Climate Survey (ICCS) which identified challenges within each domain along with evidence-based transformative and actionable strategic approaches. Reflecting the domain of Teaching and Learning, Porter (University of Manitoba) will describe a new micro-certificate program, Facilitating Older Adult Learning, that provides educators with pedagogical design tools to select and evaluate appropriate learning strategies for supporting effective older adult learning. Reflecting the domain of Student Affairs, Galucia and Morrow-Howell (Washington University in St. Louis) then will describe their research with admissions, career services, and DEI staff about how to best support non-traditional aged students. They will also discuss Next Move, a program for graduate students returning to school after substantial work and life experiences. Reflecting the domain of Personnel, Kaskie (University of Iowa) will discuss the aging professorate and how institutions can modify human resource policies and programs to provide more opportunities for aging faculty and staff to remain healthy and productive. Halvorsen (Boston College) will serve as discussant. Age Inclusivity in Higher Education Interest Group Sponsored Symposium

